# The effect of antiplatelet and anticoagulant therapies on clinical outcome of patients undergoing decompressive craniectomy: a systematic review

**DOI:** 10.3389/fneur.2024.1336760

**Published:** 2024-02-07

**Authors:** Chiara Angelini, Pietro Zangrossi, Giorgio Mantovani, Michele Alessandro Cavallo, Pasquale De Bonis, Alba Scerrati

**Affiliations:** ^1^Department of Neurosurgery, Sant’Anna University Hospital, Ferrara, Italy; ^2^Department of Translational Medicine, University of Ferrara, Ferrara, Italy; ^3^Minimally Invasive Neurosurgery Unit, Ferrara University Hospital, Ferrara, Italy

**Keywords:** antiplatelets, anticoagulants, blood thinners, decompressive craniectomy, antithrombotics

## Abstract

**Objective:**

This systematic review aims to investigate a potential correlation between the administration of antiplatelets (APs) or anticoagulants (ACs) and perioperative complications, with a particular focus on hemorrhagic events, in patients undergoing decompressive craniectomy (DC). Additionally, the secondary objective is to assess the neurological outcomes in patients undergoing DC while taking APs/ACs, comparing them to patients not on APs/ACs.

**Methods:**

The study utilized PubMed and Science Direct as primary online medical databases for the systematic review. Articles underwent screening based on title, abstract, and full-text review. Four studies meeting the inclusion criteria were selected for comprehensive analysis.

**Results:**

Our findings suggest that the administration of APs/ACs in patients undergoing DC does not significantly impact functional outcomes. Notably, the occurrence of rebleeding within 6 months and other complications, including infections, appears to be less frequent in patients taking APs compared to those not taking APs/ACs.

**Conclusion:**

Literature-derived data on the association between APs/ACs and DC presented considerable heterogeneity and insufficient volume for robust statistical analysis. Consequently, a definitive conclusion regarding the influence of suspending or continuing these therapies on complications and clinical outcomes cannot be confidently reached at present. To address this, a large-scale prospective study is warranted to gather substantial and precise data, facilitating a nuanced understanding of how to balance the risks and benefits associated with antiplatelet and anticoagulant agents in the context of decompressive craniectomy.

## 1 Introduction

The aging of the global population is an unequivocal phenomenon witnessed in recent decades, with an escalating number of individuals harboring a history of cerebrovascular or cardiovascular diseases. This has paralleled a surge in the demand for antiplatelet (APs) and anticoagulant (ACs) therapies. Concurrently, the population requiring APs/ACs and undergoing noncardiac-related surgeries has witnessed a notable increase (1).

Decompressive craniectomy (DC) is a pivotal life-saving procedure for the release of otherwise unmanageable elevated intracranial pressure (2). While frequently employed in traumatic brain injury and malignant cerebral infarction, DC’s utility extends to various pathologies, including subarachnoid hemorrhage, non-traumatic hypertensive and idiopathic cytopenic purpura-related intracranial hemorrhage (3), cerebral venous thrombosis (4, 5), infectious encephalitis (6, 7), subdural empyema (8), among others.

However, this life-saving surgical intervention is accompanied by a considerable incidence of complications (9). The three most recurrent complications encompass hemorrhagic events, infectious/inflammatory manifestations, and disturbances in the cerebrospinal fluid compartment (9). Consequently, neurosurgeons routinely grapple with the management of patients necessitating an emergent decompressive craniectomy while those patients are concurrently prescribed APs/ACs, navigating the complex decision of whether to interrupt or continue these therapies.

The perioperative management of antithrombotic agents poses formidable challenges, given the potential risks of perioperative bleeding and thromboembolic complications (1). Strikingly, to date, no established guidelines on the management of APs/ACs in patients undergoing DC have been formulated (10). The scientific literature on this topic exhibits heterogeneity, with divergent results: some studies indicate no correlation between APs/ACs usage and a heightened rate of complications, while others report an increased incidence of hemorrhagic and/or thrombotic complications in patients on these therapies compared to their counterparts without.

This review seeks to provide a comprehensive synthesis of the current scientific literature, aiming to investigate any potential correlation between APs/ACs and perioperative complications in patients subjected to DC, with a specific focus on hemorrhagic and thrombotic events. A secondary objective involves the evaluation of neurological outcomes in patients undergoing DC, differentiating those taking APs/ACs from those who are not. Ultimately, the review offers valuable insights that aim to guide the intricate perioperative management decisions surrounding the suspension or continuation of APs/ACs in neurosurgical patients undergoing decompression.

## 2 Methods

### 2.1 Study design

**Figure 1:**
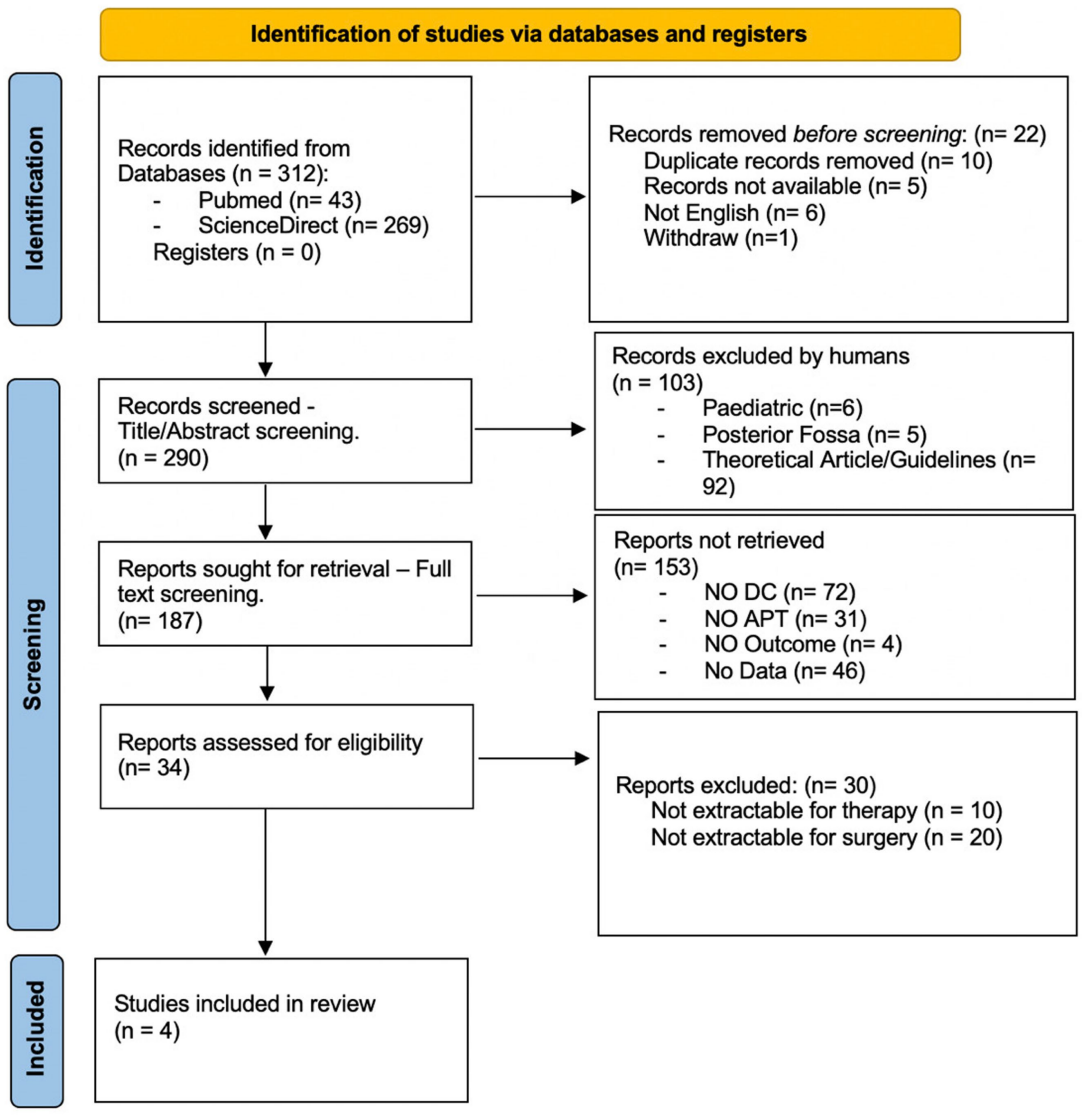
Prisma flow chart: flow of information through systematic review. DC, decompressive craniectomy; APT, antiplatelet therapy.

This systematic review is reported in accordance with the Preferred Reporting Items for Systematic Reviews and Meta-Analyses (PRISMA) (11, 12) (Figure 1).

### 2.2 Search strategy

PubMed, Ovid MEDLINE, Ovid EMBASE, Scopus, and the Web of Science were selected as online medical databases to conduct the present systematic review. The last search was launched in July 2023.

The review question was formulated according to the PICO criteria, as follows: (P, patients) patients taking anticoagulants or antiplatelet drugs, (I, intervention) undergoing decompressive craniectomy, (C, comparison) if compared to patients not taking these drugs, (O, outcomes) is the outcome worse in terms of disability and complications?

The search terms used were: “decompressive craniectomy AND (aspirin OR antiplatelet OR acetylsalicylic acid OR anticoagulant).”

### 2.3 Study selection

After removing duplicates, two authors (PZ, CA) independently identified the potentially relevant studies after reading the title, abstract, and full article text. The same authors assessed the full texts of all trials using the eligibility criteria for inclusion. Disagreements were solved through discussion or, if necessary, in consultation with a third reviewer (AS).

### 2.4 Data extraction

The extraction and analysis of data were independently performed by two authors (PZ, CA).

The patient demographic and study data extracted included year of publication, age at presentation, sex, and mean follow-up duration. Clinical data included: comorbidities, drugs, preoperative clinical condition, laboratory tests, hematoma characteristics, time of surgery from admission, type of decompressive craniectomy (frontotemporal or bifrontal), intraoperative drugs administration, postoperative conditions up to 72 h after DC, postoperative hemorrhagic complications, thromboembolic complication, and favorable or unfavorable functional outcome at 6 months.

### 2.5 Eligibility criteria

The selection criteria for this study were grounded in the objective of identifying research articles containing raw data pertaining to patients who underwent decompressive craniectomy. Where feasible, the stratification of data was conducted based on the underlying pathology and the preoperative use of antiplatelet and anticoagulant medications.

The inclusion criteria comprised:

1. Adult patients (aged over 18 years) who underwent DC following brain trauma, cerebral hemorrhage, or ischemic stroke.
2. A subset of the studied population were administered APs or ACs before DC.
3. Accessible data on the group of patients taking APs or ACs and the group not taking APs/ACs.
4. Complete scientific papers written in English.

The exclusion criteria entailed:

1. Absence of DC in the study.
2. Absence of APs or ACs use in any subset of the study.
3. Lack of reported outcome scales during the follow-up period.
4. Absence of information regarding the described complications

## 3 Results

The database inquiry using the previously delineated keywords resulted in a total of 312 studies, comprising 43 from PubMed and 269 from ScienceDirect. Prior to screening, 10 duplicated, 5 inaccessible, 6 non-English, and one withdrawn record were eliminated. After the title and abstract were screened, 103 papers were excluded, and after the full article was read, an additional 153 papers were deemed irrelevant to the aims and scopes of the research. Furthermore, 30 articles were excluded due to non-extractable data. Ultimately, four studies were incorporated into this systematic review (Table 1).

**Table 1:** Main selected papers.

First author	Year	Type of paper
Schuss P.	2013	Retrospective observational
Han H.	2016	Retrospective observational
Song X.	2016	Retrospective observational
Kinoshita T.	2020	Retrospective cohort

The paper selection process is depicted in Figure 1, with the included articles detailed in Table 1.

### 3.1 Decompressive craniectomy, APs, ACs

Four articles were ultimately selected, each providing data on the percentage of patients taking Aps or ACs, totaling 345 patients who underwent decompressive craniectomy (DC). These patients underwent decompressive craniectomy for various reasons: 86 for acute subdural hemorrhage, 43 for traumatic intracerebral hemorrhage, 101 for spontaneous intracerebral hemorrhage, 83 for ischemic stroke, and 32 for hemorrhagic stroke. Among the patients, 92 were receiving APs, 9 were receiving ACs, 3 were receiving dual antiplatelet therapy (DAPT), while 241 were not receiving any medication (refer to Table 2).

**Table 2:** Type of medications.

Medications	In all patients
APT monotherapy	83
DAPT	12
APT total	95
ACT	9
Combined therapy	0
NO APT/ACT	241
Antithrombotics	104
Total	345

### 3.2 Functional outcome (GOS/mRS)

The functional outcome at 6 months was assessed using the Glasgow Outcome Scale (GOS) or modified Rankin Scale (mRS). A favorable outcome corresponds to GOS 4–5 or mRS 0–3, while an unfavorable outcome corresponds to GOS 1–3 or mRS 4–6. Functional outcome data were available for a total of 133 patients undergoing DC (refer to Table 3).

**Table 3:** Clinical outcome at 6 months.

Outcome—6 months	In all patients	In APT/ACT	In no APT/ACT	N/A
Unfavorable outcome: GOS (1–3) mRS (4–6)	85	12	38	35
Favorable outcome: GOS (4–5) mRS (0–3)	48	10	34	4
N/A	212	63	149	-
Total	345	85	221	39

Patients not taking APs or ACs presented a favorable outcome in 47% (34 out of 72) of cases and an unfavorable outcome in 53% of cases (38 out of 72). Patients taking APs/ACs exhibited a favorable outcome in 45% (10 out of 22 patients) and an unfavorable outcome in 55% of cases (12 out of 22 patients). Data regarding functional outcomes were not available for 212 patients.

### 3.3 Rebleeding

The rebleeding rate before and at 6 months was assessed during follow-up, considering the type of hematoma, localization, chronological distribution of bleeding, and the need for reintervention. Among all patients subjected to DC, including those taking APs or ACs and those not, rebleeding occurred in 108 before 6 months and in an additional 2 at 6 months. Of these 108 patients experiencing rebleeding before 6 months, 35% belonged to the APs/ACs group (38 out of 108), and 48% to the group of patients not taking APs or ACs (52 out of 108). Further details are provided in Table 4.

**Table 4:** Bleeding complications.

Bleeding complications	In all patients	In APT/ACT	In no APT/ACT	N/A
Rebleeding before 6 months	108	38	52	18
Not rebleeding	237	47	169	21
Total	345	85	221	39

### 3.4 Other complications

Regarding non-hemorrhagic complications during convalescence, thromboembolic events (stroke, heart attack, deep vein thrombosis, cardiopulmonary failure), and other complications such as infections were documented. Data on non-hemorrhagic complications were available for 216 patients. Among all patients, the most frequent complication was infection, occurring in 74 patients: 23 in the APs/ACs group (31%) and 51 in the group not taking APs or ACs (69%). Other complications occurred in 37 patients, with 11 in the APs/ACs group (30%) and 26 in the group not taking APs or ACs (70%). Stroke occurred in 11 patients overall, with 3 taking APs/ACs (27%) and 8 not taking them (73%). Further details are reported in Table 5.

**Table 5:** Other complications.

Other complications (different from bleeding)	In all patients	In APT/ACT	In no APT/ACT
Stroke	11	3	8
Heart attack	0	0	0
Deep vein thrombosis	0	0	0
Infections	74	23	51
Cardiopulmonary failure	0	0	0
Others	37	11	26
N/A	129	38	91
Total of complications	122	37	85
Total of patients	345	104	241

Data on the type of pathology and the type of medications are outlined in Table 6.

**Table 6:** Type of pathology.

Type of pathology	In all patients
Trauma aSDH	86
Trauma EDH	0
Trauma SAH	0
Trauma ICH	43
Spontaneous ICH	101
Ischemic stroke	83
Hemorrhagic stroke	32
N/A	0
Total	345

## 4 Discussion

The data analysis process involved the stratification of available data into distinct subgroups based on factors such as age, underlying pathology, comorbidities, and preoperative clinic. However, the endeavor to establish meaningful subgroups was hindered by the insufficiency and heterogeneity of the available data.

Our observations may indicate that the use of ACs or APs does not alter functional outcomes. Notably, the majority of patients exhibited an unfavorable outcome, irrespective of APs/ACs intake.

Regarding hemorrhagic complications, our data reveal a more consistent occurrence of rebleeding before 6 months in patients not taking APs or ACs (48% vs. 35% in patients taking APs/ACs).

Concerning non-hemorrhagic complications, the infection rate was higher in patients not taking APs or ACs (69% vs. 31% in patients taking APs/ACs), as well as ischemic cerebrovascular insults (73% in patients not taking APs or ACs vs. 27% in patients taking APs/ACs) and other complications (70% in patients not taking APs or ACs vs. 30% in patients taking APs/ACs).

These data imply that patients undergoing DC are vulnerable, exhibiting a higher likelihood of unfavorable outcomes or increased complication rates, irrespective of ACs or APs administration. While these treatments may suggest a higher frailty, such as the presence of cardiovascular diseases, they do not appear to significantly influence the ultimate outcome.

Han et al. (13) conducted a retrospective analysis involving 90 patients with TBI who underwent emergent DC. Nineteen of these patients were using antiplatelet agents before TBI. The incidence of hemorrhagic complications was 52.6% (10 out of 19) in group 1 and 46.5% (33 out of 71) in group 2 (*p* = 0.633). The reoperation rate was 36.8% (7 out of 19) in group 1 and 36.6% (26 out of 71) in group 2 (*p* = 0.986). No statistically significant difference was observed between the two groups.

In a retrospective observational study by Schuss et al. (14) data were collected from 115 patients who underwent decompressive craniectomy due to acute ischemic stroke. They compared patients with and without intravenous thrombolysis (IVT) before DC, assessing functional outcomes at 3 months using the mRS, along with bleeding and other complications. Forty-four patients out of 115 were on antiplatelet therapy before DC (38%). The study concluded that bleeding complications occurred significantly more frequently in patients with antiplatelet use before DC (*p* = 0.0003). In the multivariate analysis, “preoperative use of acetylsalicylic acid” emerged as the only independent predictor associated with bleeding complications (*p* = 0.002). The use of intravenous thrombolysis was suggested to have a more pronounced bleeding effect compared to standard ACs or APs.

Lastly, Kinoshita et al. (15) conducted a retrospective cohort study involving 91 patients with TBI undergoing evacuation of intracranial hemorrhagic lesions. The preoperative use of APs and ACs was also assessed. Regarding outcomes at 6 months and delayed hemorrhage, the study’s findings did not indicate a discernible distinction between patients undergoing decompressive craniectomy taking APs/ACs and those not taking these therapies.

Schuss et al. (14) was the only study among the four selected that reported a relapse of antiplatelets on rebleeding complications. In contrast, Han et al. (13) and Song et al. (16) reported that the rate of hemorrhagic complications and reoperation was not affected by APs/ACs.

### 4.1 Limitations

As previously noted, a primary limitation of this review stems from the paucity of available data in the literature. The inadequacy of data precludes a robust statistical analysis, particularly due to the infrequency with which the association between patients undergoing DC and their potential use of APs or ACs is explored. This current article highlights a significant limitation stemming from the heterogeneous nature of available literature data, presenting challenges in several key aspects. Notable instances of this heterogeneity include the amalgamation of antiplatelet and anticoagulant therapies in numerous studies, despite their distinct pharmacodynamic properties and specific indications. Another crucial aspect is the non-separation of patients undergoing craniotomy and craniectomy surgeries in certain studies, overlooking profound differences in indications, pathology severity, post-surgical complications, and postoperative days in the intensive therapy department. Many investigations on DC outcomes fail to exclusively focus on DC, often encompassing a broader population, leading to data that apply to the entire cohort rather than specifically to those undergoing DC. The incomplete reporting of clinical elements, both pre-and post-operatively, is noted as a significant observation, impeding effective patient stratification. Additionally, some studies either do not report postoperative outcomes or provide data that is challenging to interpret due to unclear definitions and temporal aspects. Lastly, a prevalent practice is the limited presentation of raw data, with many studies synthesizing data without offering access to the raw information, compromising the transparency and interpretability of the findings. Additionally, Kinoshita et al. (15) concentrated on an elderly population, restricting their research to patients aged 60 years or older. Considering this age group within the context of a highly fatal underlying disease introduces a potential bias. Furthermore, none of the studies have investigated strategies for managing APs/ACs or the timing of their interruption, meaning definitive conclusions about the strategy and timing for managing APs/ACs cannot be derived.

### 4.2 Future perspectives

To address the identified limitations, a comprehensive prospective observational study is imperative, encompassing detailed data on patients undergoing DC and their use of APs/ACs. This study should additionally evaluate the initiation and discontinuation times of these therapies. Such an investigation is essential for informing daily practice and guiding surgeons on the optimal management of antiplatelet and anticoagulant therapies in patients subjected to DC.

## 5 Conclusion

Although our results tentatively suggest that the use of APs/ACs in patients undergoing DC may not significantly impact the final functional outcome, the occurrence of rebleeding before 6 months and other complications, such as infections, appears to be less frequent. Moreover, the considerable heterogeneity of the data precludes the formulation of definitive guidelines regarding the management of antiplatelet and anticoagulant therapies in patients undergoing DC. A comprehensive prospective observational study, coupled with an initiative within the scientific community to standardize data reporting methods in neurotrauma articles, is essential. This effort aims to gather sufficient and accurate data, facilitating a nuanced understanding of how to balance the risks and benefits associated with antiplatelet and anticoagulant agents in the context of DC.

## Data availability statement

The original contributions presented in the study are included in the article/supplementary material, further inquiries can be directed to the corresponding author.

## Author contributions

CA: Writing – original draft. PZ: Writing – original draft. GM: Writing – review & editing. MC: Writing – review & editing. PB: Writing – review & editing. AS: Writing – review & editing.

## References

[1] Fujikawa T Kawamura Y Takahashi R Naito S. Risk of postoperative thromboembolic complication after major digestive surgery in patients receiving antiplatelet therapy: lessons from more than 3,000 operations in a single tertiary referral hospital. Surgery. (2020) 167:859–67. doi: 10.1016/j.surg.2020.01.003 32087945

[2] Sturiale CL De Bonis P Rigante L Calandrelli R D’Arrigo S Pompucci A . Do traumatic brain contusions increase in size after decompressive craniectomy? J Neurotrauma. (2012) 29:2723–6. doi: 10.1089/neu.2012.2556 22873699

[3] Ranger A Szymczak A Fraser D Salvadori M Jardine L. Bilateral decompressive craniectomy for refractory intracranial hypertension in a child with severe ITP-related intracerebral haemorrhage. Pediatr Neurosurg. (2009) 45:390–5. doi: 10.1159/000260910, PMID: 19940538

[4] Aaron S Alexander M Moorthy RK Mani S Mathew V Patil AKB . Decompressive craniectomy in cerebral venous thrombosis: a single Centre experience. J Neurol Neurosurg Psychiatry. (2013) 84:995–1000. doi: 10.1136/jnnp-2012-303356, PMID: 23591554

[5] Ferro JM Crassard I Coutinho JM Canhão P Barinagarrementeria F Cucchiara B . Second international study on cerebral vein and Dural sinus thrombosis (ISCVT 2) investigators decompressive surgery in cerebrovenous thrombosis: a multicenter registry and a systematic review of individual patient data. Stroke. (2011) 42:2825–31. doi: 10.1161/STROKEAHA.111.615393, PMID: 21799156

[6] Adamo MA Deshaies EM. Emergency decompressive craniectomy for fulminating infectious encephalitis. J Neurosurg. (2008) 108:174–6. doi: 10.3171/JNS/2008/108/01/0174, PMID: 18173329

[7] Pérez-Bovet J Garcia-Armengol R Buxó-Pujolràs M Lorite-Díaz N Narváez-Martínez Y Caro-Cardera JL . Decompressive craniectomy for encephalitis with brain herniation: case report and review of the literature. Acta Neurochir. (2012) 154:1717–24. doi: 10.1007/s00701-012-1323-3, PMID: 22543444

[8] Ong YK Goh KYC Chan C. Bifrontal decompressive craniectomy for acute subdural empyema. Childs Nerv Syst. (2002) 18:340–3. doi: 10.1007/s00381-002-0597-9 12172943

[9] Kurland DB Khaladj-Ghom A Stokum JA Carusillo B Karimy JK Gerzanich V . Complications associated with decompressive craniectomy: a systematic review. Neurocrit Care. (2015) 23:292–304. doi: 10.1007/s12028-015-0144-7, PMID: 26032808 PMC4704457

[10] Lewis SR Pritchard MW Schofield-Robinson OJ Alderson P Smith AF. Continuation versus discontinuation of antiplatelet therapy for bleeding and ischaemic events in adults undergoing non-cardiac surgery. Cochrane Database Syst Rev. (2018) 1:CD012584. doi: 10.1002/14651858.CD012584.pub2 PMC6513221 30019463

[11] Moher D Liberati A Tetzlaff J Altman DG PRISMA Group. Preferred reporting items for systematic reviews and meta-analyses: the PRISMA statement. PLoS Med. (2009) 6:e1000097. doi: 10.1371/journal.pmed.1000097 19621072 PMC2707599

[12] Page MJ McKenzie JE Bossuyt PM Boutron I Hoffmann TC Mulrow CD . The PRISMA 2020 statement: an updated guideline for reporting systematic reviews. BMJ. (2021) 372:n71. doi: 10.1136/bmj.n71 33782057 PMC8005924

[13] Han H Koh EJ Choi H Kim BC Yang SY Cho KT. The effect of preoperative antiplatelet therapy on hemorrhagic complications after decompressive Craniectomy in patients with traumatic brain injury. Korean J Neurotrauma. (2016) 12:61–6. doi: 10.13004/kjnt.2016.12.2.61, PMID: 27857909 PMC5110920

[14] Schuss P Borger V Vatter H Singer OC Seifert V Güresir E. Antiplatelet therapy, but not intravenous thrombolytic therapy, is associated with postoperative bleeding complications after decompressive craniectomy for stroke. J Neurol. (2013) 260:2149–55. doi: 10.1007/s00415-013-6950-y 23712799

[15] Kinoshita T Yoshiya K Fujimoto Y Kajikawa R Kiguchi T Hara M . Decompressive craniectomy in conjunction with evacuation of intracranial hemorrhagic lesions is associated with worse outcomes in elderly patients with traumatic brain injury: a propensity score analysis. World Neurosurg. (2016) 89:187–92. doi: 10.1016/j.wneu.2016.01.071, PMID: 26851740

[16] Song X Zhang Q Cao Y Wang S Zhao J. Antiplatelet therapy does not increase mortality of surgical treatment for spontaneous intracerebral haemorrhage. Clin Neurol Neurosurg. (2020) 196:105873. doi: 10.1016/j.clineuro.2020.105873 32531616

